# Preconditioned Mesenchymal Stromal Cells to Improve Allotransplantation Outcome

**DOI:** 10.3390/cells10092325

**Published:** 2021-09-06

**Authors:** Hui-Yun Cheng, Madonna Rica Anggelia, Cheng-Hung Lin, Chih-Fan Lin

**Affiliations:** 1 Center for Vascularized Composite Allotransplantation, Linkou Chang Gung Memorial Hospital, Taoyuan 333, Taiwan; lukechlin@gmail.com (C.-H.L.); free4ever2go@gmail.com (C.-F.L.); 2 Department of Plastic and Reconstructive Surgery, Linkou Chang Gung Memorial Hospital, Taoyuan 333, Taiwan; mranggelia@yahoo.com; 3 School of Medicine, Chang Gung University, Taoyuan 333, Taiwan

**Keywords:** mesenchymal stromal cells, allotransplantation, preconditioning

## Abstract

Mesenchymal stromal cells (MSCs) are tissue-derived progenitor cells with immunomodulatory as well as multilineage differentiation capacities, and have been widely applied as cellular therapeutics in different disease systems in both preclinical models and clinical studies. Although many studies have applied MSCs in different types of allotransplantation, the efficacy varies. It has been demonstrated that preconditioning MSCs prior to in vivo administration may enhance their efficacy. In the field of organ/tissue allotransplantation, many recent studies have shown that preconditioning of MSCs with (1) pretreatment with bioactive factors or reagents such as cytokines, or (2) specific gene transfection, could prolong allotransplant survival and improve allotransplant function. Herein, we review these preconditioning strategies and discuss potential directions for further improvement.

## 1. Introduction

The allotransplantation of the corresponding organ or tissue(s) has become a powerful therapeutic strategy for patients with terminal illness or severe defects in form or function. However, the immunological barrier between the donor and the recipient is difficult to concur. Currently, the long-term success of allotransplantation mainly relies on the constant intake of immunosuppressant drugs; however, along with their benefits in preventing rejection, immunosuppressant drugs may result in potentially serious adverse effects, such as drug toxicities, opportunistic infections, and potentially fatal malignancies [1]. To minimize these adverse effects, alternative or adjunctive strategies are being explored. To this end, the application of cellular therapeutics with immunomodulatory properties has attracted much attention.

It was summarized that the characteristics of suitable cellular therapeutics include their (1) potential to be acquired in appropriate therapeutic amount by harvesting from the donor source, or in vitro expansion, (2) capability to survive the cryopreservation condition, and (3) safe route of administration [2]. Allogenecity should be considered if the cells are derived from sources other than the recipient origins [2]. Mesenchymal stromal cells (MSCs) meet all of these criteria and have great promise for application as cellular therapeutics.

MSCs are heterogeneous progenitor cells that exhibit the potential of self-renewal and multilineage differentiation. According to the International Society for Cell and Gene Therapy (ISCT), the criteria of MSCs include plastic adherence, positive expression of specific cluster of differentiation (CD) molecules CD73, CD90, and CD105, negative expression of hematopoietic and endothelial markers and human leukocyte antigen DR (HLA-DR), and in vitro differentiation capacities into adipocyte, chondrocyte, and osteoblast lineages [3]. Although first identified in bone marrow in 1968, MSCs are now known to exist in essentially all kinds of tissues [4], and the tissue source influences the properties of these cells [5]. The ISCT recommends that the tissue source be affixed to the MSCs; for example, BM-MSCs and AD-MSCs represent MSCs isolated from the bone marrow (BM) and adipose tissue, respectively [3].

In vivo and in vitro studies have demonstrated the potent immunomodulatory capacities of MSCs toward several immune cell populations [6]. For example, MSCs are shown to suppress the proliferation of effector T (Teff), B, and natural killer (NK) cells, whereas they promote the production of regulatory T cell (Treg) and differentiation of the regulatory-type M2 macrophages. MSCs also affect the differentiation of dendritic cells (DCs) to their tolerogenic phenotype, which have the properties of lower antigenicity, higher production of IL-10, and lower secretion of IL-12. MSCs may exert their functions through cell–cell contact or, as trophic mediators, by secreting bioactive factors that have immunoregulatory or anti-inflammatory functions, such as prostaglandin E2 (PGE_2_) and indoamine-2, 3-dioxygenase (IDO), heme oxygenase-1 (HO-1), interleukin-10 (IL-10), and transforming growth factor beta (TGF-β) [7].

Through their pro-angiogenesis and anti-apoptotic properties, MSCs enhance tissue repair by secreting factors such as vascular endothelial growth factor, hepatocyte growth factor (HGF), and angiopoietin-1, and by activating the prosurvival Akt signaling pathway [8]. Furthermore, MSCs may respond to mitochondrial damage-associated molecular patterns (DAMPs) in an inflammatory environment by transferring mitochondria to damaged cells for tissue repair [9]. It is also demonstrated recently that MSCs may modulate the CD4^+^ T cells and Tregs through direct mitochondria transfer [10,11]. Allotransplantation inherently elicits ischemia–reperfusion injuries during surgery and alloantigen-induced immune responses including proliferation of lymphocytes and secretion of cytokines. The above-mentioned characteristics of MSCs make them ideal cellular therapeutics for allotransplantation.

## 2. MSCs in Allotransplantation and Limitations

MSCs have been applied in various forms of organ and tissue allotransplantation. In animal studies, MSCs induced donor-specific tolerance in organ and vascularized composite allotransplantation (VCA) [12,13,14]. Over the past 10 years, many clinical studies have been performed on the application of MSCs in organ transplantation patients. Due to variations in the sources, doses, administration routes, timing, and frequencies of MSCs and the accompanied immunosuppression regimen, a definite conclusion on the benefits of MSCs in allotransplantation is yet to be achieved. For example, several studies have shown that the infusion of MSCs to transplantation recipients is safe [15,16,17]; however, engraftment syndrome and up to 50% opportunistic infection have also been reported [18,19]. Some studies did not report significant improvements in acute rejection occurrence and graft function after MSC administration [16,20]. On the other hand, Tan et al. reported that MSC administration enabled lower dose of calcineurin inhibitor (CNI) immunosuppressants, without compromising graft function or acute rejection occurrence [21]. Reinders et al. recently demonstrated that MSCs infused at 6 and 7 weeks after kidney transplantation allowed the withdrawal of tacrolimus without deterioration in graft function or increase in rejection rates [22]. Although these data provided a great promise for the application of MSCs as cellular therapeutics to improve the allotransplantation outcomes, discrepancies between studies imply the scope for optimization, which can be considered in the process of MSC preparation and administration.

Generally, MSCs are isolated from tissues by enzymatic digestion and selection, followed by growth in rich cell culture media and proliferation before administration back into the in vivo environment. The in vivo environment is different from the in vitro culture condition, in terms of its lower oxygen supply and associated proinflammatory milieu frequently. Such environment may cause stress to the MSCs. For example, it was estimated that <1% MSCs survived for 4 days after intracardiac injection [23]. Furthermore, with the most common intravenous (IV) administration, imaging studies revealed that >80% MSCs accumulated in the lung vasculature within minutes after infusion, followed by their appearance in the liver and spleen [24]. The MSC-derived signal had a half-life of 24 hours and disappeared within days [25]. In the meantime, only a small proportion of cells responded to chemotaxis signals released by the tissues and migrated to the target destination through mechanisms similar to leukocyte migration mediated by integrins and adhesion molecules [26]. Another aspect less discussed is the metabolic state of the MSCs. In quiescence (such as in the bone marrow), MSCs are majorly glycolytic with active mitophagy and autophagy. When MSCs grow in nutrient-rich culture conditions for expansion, they shift to rely on oxidative phosphorylation for rapid proliferation. Many cytotoxic byproducts such as reactive oxygen species (ROS) and senescent cells are then generated, and consequentially affect the therapeutic efficacy [27]. Thus, the therapeutic efficacy of MSCs can be potentially enhanced by extending MSC survival, controlling MSC metabolism, and facilitating MSC homing within the recipient body.

## 3. Improving MSC Efficacy by Preconditioning

**Figure 1:**
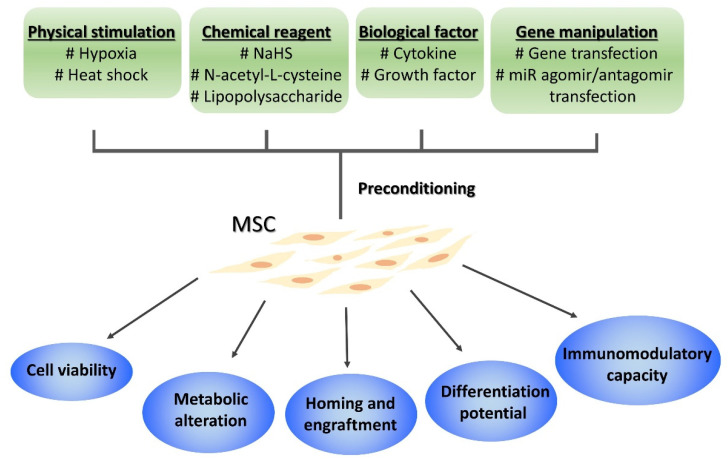
Preconditioning strategies of MSCs and the elicited effects. Only some examples in each category are listed.

Over the years, preconditioning MSC before in vivo administration has been performed to improve efficacy of MSC therapy. For example, Mangi et al. demonstrated that BM-MSCs transfected with the prosurvival gene Akt1 showed lower apoptosis rate and higher degree of tissue retention following their transplantation into ischemic myocardium; Akt1-transfected MSCs also showed significantly better efficacy to reduce infarct volume and restore the cardiac function than their non-transfected counterparts [28]. Many preconditioning strategies, including modification of physical environment, chemical/pharmaceutical reagents, biological factors, and gene manipulation, have been applied to MSCs; these strategies affect MSCs in various aspects [29,30] (Figure 1). In consequence, preconditioned MSCs have shown improved therapeutic efficacy in different disease models, such as myocardial infarction [31], brain injury [32], colitis [33], and graft-versus host disease [34].

Incorporating preconditioned MSCs, outcome of the organ/tissue allotransplants, including the heart, liver, intestine, and kidney organ, as well as corneal, skin, and composite tissues such as hindlimb (a form of VCA) can be improved (Table 1). Herein we will review these recent studies of preconditioned MSCs applied in organ/tissue allotransplantation. By doing so, we hope to find the critical factor(s) that affect the MSC efficacy and then acquire strategies to further promote allotransplant survival, reduce the reliance of traditional immunosuppressants, and hopefully induce donor-specific tolerance. The preconditioning strategies currently applied in transplantation studies can be roughly divided into pretreatment by reagents/biological factors and by gene transfection and will be discussed below. For clarification purposes, in this report, the MSCs are labeled by the pretreatment reagent to the right and the transfected genes to the left; for example, Akt1-MSC and MSC^IL-17^ represent Akt1-transfected and IL-17-pretreated MSCs, respectively.

### 3.1. Pretreatment

#### 3.1.1. Cytokines IFN-γ, TNF-α, IL-1β, IL-17, TGF-β

The immunomodulatory capacity of MSCs could be enhanced upon exposure to proinflammatory cytokines such as interferon gamma (IFN-γ) or IL-1β [30,35]. Murphy et al. demonstrated that MSCs pretreated with proinflammatory cytokines tumor necrosis factor alpha (TNF-α) and IL-1β significantly suppressed T cell proliferation induced by anti-CD3/CD28, mainly through nitric oxide (NO) and PGE_2_ [36]. When applied to corneal allotransplantation, MSCs^TNF-^^α/IL-1β^ and untreated MSCs prolonged allograft survival in 70% and 50% recipients, respectively. These effects were associated with the production of NO by the MSCs^TNF-^^α/IL-1β^ because the transfection of iNOS shRNA to MSCs^TNF-^^α/IL-1β^ abolished the benefits on allotransplant survival. Furthermore, MSCs^TNF-^^α/IL-1β^, instead of MSCs, induced persistent elevation of CD4^+^CD25^+^FoxP3^+^ Tregs in the lung and spleen distant from the transplant. IV-infused MSCs^TNF-^^α/IL-1β^ induced regulatory myeloid CD11b^+^MHC II^+^B220^+^ cells (MHC stands for major histocompatibility complex) in the lung, which highly induced PGE_2_ and further promoted Treg generation. These data suggest that compared with untreated MSCs, MSCs^TNF-^^α/IL-1β^ instructed a more potent immunomodulatory function. Similarly, compared with untreated BM-MSCs, BM-MSCs^IFN-^^γ^ exhibited potentiated suppression in T cell proliferation and also significantly prolonged VCA survival with elevated spleen Treg levels. In this system, IFN-γ exerted its function through enhancing programmed death-ligand 1 (PD-L1) expression, and the effects of BM-MSC^IFN-^^γ^ were offset by the PD-L1 antibody. Interestingly, the response of BM-MSCs to IFN-γ was in a bell shape, such that 10 ng/mL showed the best efficacy within the test concentration range of 5–30 ng/mL [37]. Ma et al. demonstrated that IL-17 pretreatment to BM-MSCs significantly prolonged grafted alloskin survival. Analysis on POD 7 demonstrated that IL-17 pretreatment promoted the homing of more MSCs to the allograft, accompanied by less proinflammatory cell infiltration and more angiogenesis. Consequently, elevation of Treg in the spleen and increased IL-10 and TGF-β levels in the circulation were observed [38].

Anti-inflammatory TGF-β has also been used as a pretreatment of MSCs, with the capacity of auto-induction to secrete of TGF-β1 and TGF-β2 by MSCs, in addition to the expression of the TGF-β receptor isoform TβRIIA [39]. Lynch et al. reported that murine BM-MSCs^TGF-β^ expressed higher levels of CD73 and lower levels of stem cell antigen-1 (SCA-1) and MHC I. BM-MSC^TGF-β^ suppressed T lymphocyte proliferation and enhanced Treg expansion through cell–cell contact and interaction of PGE_2_-EP4; compared with untreated MSCs, they also showed increased production of PGE_2_, secreted forms of TNF receptor, and had lower levels of TNF-α, IFN-γ, and IL-6. When applied to murine corneal allotransplantation, BM-MSCs^TGF-β^ significantly prolonged allograft survival compared with untreated MSCs. They also significantly reduced CD4^+^ and CD8^+^ effector T cells in draining lymph nodes (DLN) and increased Tregs in DLN and the lung. Furthermore, the frequency of and expression of costimulatory molecules of DCs were suppressed [40]. The data did not support TGF-β auto-induction as the major mechanism of the benefits provided by BM-MSC^TGF-β^, suggesting different doses of TGF-β (3 ng/mL vs. 50 ng/mL) and cell origin (human AD-MSC vs. mice BM-MSC) had impacts on the downstream effects that TGF-β may inflict on MSCs.

#### 3.1.2. Toll-Like Receptor, TLR3 Agonist Poly(I:C)

Toll-like receptors (TLRs) contribute to the innate immune system by interacting with pathogen- or damage-associated molecular patterns. Activation of TLR3 and TLR4 can polarize MSCs to their anti- and proinflammatory subtypes MSC2 and MSC1, respectively [41]. Treatment of AD-MSCs with the TLR3 agonist poly(I:C) enhanced their suppressive potential against CD4^+^ cell proliferation in vitro. Poly(I:C) specifically increased the gene and protein expression of the Treg target molecule fibronectin-like protein 2 (Fgl2), rather than PGE_2_ or IL-10, suggesting that Fgl2 is the main effector. Infusion of AD-MSCs^poly(I:C)^ promoted heterotopic cardiac allograft survival, which was accompanied by elevation of CD4^+^FoxP3^+^ Treg in the spleen on POD 4. Both in vitro and in vivo data suggested that AD-MSC^poly(I:C)^ exerted the immunomodulatory effects through Tregs. Histological studies of the transplanted heart also supported the protective effects of AD-MSCs^poly(I:C)^. No additive effects of poly(I:C) and the TLR4 antagonist TAK242 on AD-MSCs were reported in this study [42].

#### 3.1.3. Erythropoietin

Erythropoietin (EPO) regulates the growth and differentiation of the erythroid progenitor cells by binding to the EPO receptor and has been widely used to treat kidney injury. EPO also demonstrated its immunomodulatory effects by promoting Treg generation and production of TGF-β by the APCs [43]. Infusion of EPO-pretreated BM-MSCs in renal transplant recipients resulted in improved renal function and significant decrease in the IFN-γ/IL-4 ratio in the transplanted renal tissue. The effects of EPO in promoting BM-MSC survival and migratory capability through upregulated C-X-C chemokine receptor type 4 (CXCR4) expression may be relevant [44].

### 3.2. Gene Transfection

#### 3.2.1. Anti-inflammatory Cytokine: TGF-β1, IL-10

IL-10 is a well-known anti-inflammatory cytokine, whose elevation was reported to affiliate with regulatory subtypes within T and B cells and macrophages (Treg, Breg, and Mreg, respectively). Both systemically [45] and locally [46] infused IL-10-BM-MSCs significantly prolonged allograft survival compared with MSCs. The IV-infused IL-10-BM-MSCs migrated to the transplanted liver within 24 hours and could be retained for 7 days. Intragraft increase of Foxp3 and decrease of retinoic acid receptor-related orphan nuclear receptor gamma (ROR-γt) were observed after POD 5, along with the elevation of IL-10 and TGF-β1 and decrease in IL-6, TNF-α, IFN-γ, IL-23, and IL-17 levels, suggesting that the modulation of Treg/Th17 axis occurred [45]. In the cornea transplantation system, subconjunctival injected IL-10-BM-MSCs resided around the injection site for at least 10 days. Less graft-infiltrated CD4^+^ and CD68^+^ cells and more Tregs in DLN were identified in recipients treated with IL-10-BM-MSCs. IL-10-BM-MSCs also upregulated long non-coding RNA (lncRNA) 003946 expression in CD68^+^ macrophages of the grafted cornea, reduced the antigen presentation capacity of macrophages, and suppressed alloimmune responses [46].

Tang et al. reported that TGF-β1-BM-MSCs induced the conversion of CD4^+^CD25^-^ cells to Tregs in vitro. Through portal vein infusion, TGF-β1-BM-MSCs prolonged the survival of transplanted liver and alleviated the severity of rejection. They promoted a local immunosuppressive microenvironment in the graft by reducing the expression of IL-6, IL-1β, TNF-α, and IFN-γ and enhancing the expression of IL-10 by POD 7. The concurrent generation of Helios^-^FoxP3^+^ iTregs and reduction in Th17 cells contributed to prolonged allograft survival [47].

#### 3.2.2. Anti-inflammatory Mediator: IDO, HO-1, HGF

MSCs exert their immunomodulatory functions partially through the production of protein factors, such as IDO, which depletes tryptophan in the local environment and blocks lymphocyte proliferation. In vitro studies showed that IDO-BM-MSCs induced Treg generation with higher expression of coinhibitory cytotoxic T-lymphocyte-associated protein 4 (CTLA-4), suppressed CD4^+^CD25^−^ effector T cell proliferation, and downregulated the expression of costimulatory molecules in cocultured DCs [48,49]. Following in vivo administration, IDO-BM-MSCs promoted donor-specific tolerance in kidney transplantation recipients and improved allograft function and structural integrity [49]. IDO-BM-MSCs exerted their effects through elevation of Tregs and anti-inflammatory IL-10 and TGF-β in the circulation, rather than through the production of PGE_2_.

HO-1 plays a critical role in metabolizing the cell-free form of heme, which is released during cell apoptosis and tissue damage. Thus, HO-1 exerts its protective activity in different disease settings such as ischemia–reperfusion injury and graft versus host disease [50]. It is also an immunomodulatory mediator that MSCs produce and employ [51]. HO-1-BM-MSCs were applied to transplantation of liver [52,53] and small bowel [54] with superior efficacy to prolong transplant survival, improve transplant function, and ameliorate rejection manifestation than the untransfected BM-MSCs. The beneficiary effects of HO-1-BM-MSC were associated with increased production of Treg in the spleen and lower levels of proinflammatory cytokines in serum. Moreover, suppression of NK cell activity and viability in the recipient spleen was observed.

HGF was originally identified as a mitogenic factor of hepatocytes and later characterized with proangiogenic, anti-apoptotic, and immunomodulatory capacities [55]. HGF was recently found to participate in the immunomodulatory effects exerted by MSCs, such as regulating the Treg/Th17 balance, inducing regulatory DCs, and reducing antigen presentation capacity of DCs through their paracrine actions [56,57]. HGF-MSCs were demonstrated to exhibit potent anti-apoptotic and proangiogenesis capacities [58]. Bian et al. demonstrated that HGF-BM-MSCs significantly prolonged the survival of allografted skin. However, they were not superior to BM-MSCs in suppressing lymphocyte proliferation. The authors suggested that the reparative properties of HGF-MSCs may have contributed to prolonged alloskin survival [59].

#### 3.2.3. Signal 2 for T Cell Activation: OX40Ig, PD-L1Ig

Complete activation of T cells requires three signals: antigen recognition (Signal 1), co-stimulation (Signal 2), and cytokine priming (Signal 3). Signal 2 is mediated by various pathways and could be either stimulatory or inhibitory to T cell activation. Manipulation of co-stimulation pathways (i.e., costimulatory blockade) by anti-CD40L, PD-L1Ig, and OX-40Ig was effective in promoting allotransplant survival and inducing donor-specific tolerance [60]. The combination of the immunomodulatory function of MSCs and costimulatory blockade by transfecting MSCs with PD-L1Ig or OX-40Ig potentiated the suppression of T cell proliferation in vitro. OX-40Ig-AD-MSCs ameliorated transplanted renal damage with lower serum creatine level and slightly prolonged the survival of the transplanted kidney. Furthermore, decreased expression of IFN-γ and increased expression of IL-10, TGF-β, and FoxP3 were observed within the graft [61]. Similarly, PD-L1Ig-BM-MSCs significantly improved the function of the transplanted liver and significantly prolonged allograft survival [62].

#### 3.2.4. Treg and Treg Effector: FoxP3, sFgl2, IL-35

Many of the above-mentioned gene-modified MSCs exerted their effects at least partially by promoting Treg generation. Transfection of BM-MSCs with Treg-specific FoxP3 further enhanced the production of Treg when cocultured with CD4^+^ T cells. FoxP3-BM-MSC suppressed alloantigen-induced CD4^+^ T cell proliferation through cell–cell contact and enhanced PD-L1 expression. FoxP3-BM-MSC administration in liver transplantation recipients induced donor-specific tolerance, which was abolished by Treg depletion by the CD25 antibody [63].

Tregs may exert immunosuppressive functions by secreting protein factors, such as IL-35 and soluble form of Fgl2 (sFgl2) [64]. IL-35 inhibits the production of Th1 and Th17 and converts naïve T cells to IL-35-secreting Tregs [65]. Infusion of IL-35-AD-MSCs in cardiac transplantation recipients significantly prolonged allograft survival, in addition to suppressing the production of Th17 within the grafted heart and spleen. Lower Th1/Th2 ratio and increased production of CD4^+^FoxP3^+^ Treg were also noted in the spleen [66].

Overexpression of sFgl2 promoted liver transplant survival by promoting M2 polarization of Kupffer cells [67]. Similarly, sFgl2-AD-MSCs promoted M2 polarization in vitro, especially in a proinflammatory environment with IFN-γ/lipopolysaccharide (LPS) treatment, through the inhibition of the signal transducer and activator of transcription 1 (STAT1) and nuclear factor-κB (NF-κB) pathways. sFgl2-AD-MSCs migrated to the transplant when administered to cardiac transplantation recipients and led to a higher intragraft M2/M1 ratio in infiltrated macrophages. M2 polarization was maintained in the spleen along with elevation of Tregs. In serum, decreased IFN-γ, TNF-α, IL-1β, IL-6, and IL-12 levels and increased IL-4, IL-10, and TGF-β1 levels were observed in sFgl2-AD-MSCs-treated recipients on POD 7 and 14 [68].

#### 3.2.5. Chemokine Receptor: CCR7, CXCR4, CXCR3

Interaction between chemokines and cognate receptors is critical for cell trafficking and homing to specific action sites. C-C motif chemokine receptor 7 (CCR7) binds to its ligand secondary lymphoid organ (SLO) ligand (SCL) and directs the transmigration of CCR7-expressing cells to the SLO, where naïve T cells encounter antigens presented by APCs. Following infusion to the VCA recipient, CCR7-AD-MSCs migrated to the T cell aggregation area in SLO and significantly delayed rejection. Alterations in the Th1/Th2 and Th17/Treg ratios in SLOs and plasma cytokine levels were observed from POD 3 [69].

The CXCR4 ligand SDF-1 is expressed abundantly in inflammatory or injured tissues, where CXCR4-MSCs are expected to migrate to. Following infusion to the kidney transplant recipients, CXCR4-MSCs were found in the transplanted kidney on POD 3, which led to improved renal function and intense FoxP3 immunostaining in the renal interstitium [70]. However, the effects on allograft survival were not reported.

Similarly, CXCR3-MSCs were hypothesized to migrate to the injury or rejection sites where the CXCR3 ligands, MIG, IP-10, and I-TAC, were abundantly expressed. Yin et al. constructed and administered CXCR3/HO-1-BM-MSCs in small intestine transplantation recipients. BM-MSCs appeared in the transplant on POD 1, with increased intragraft expression of CXCR3 and HO-1. Less intragraft apoptotic cells and lower NK cell activity were observed, accompanied by elevation of spleen Tregs. CXCR3/HO-1-BM-MSCs significantly prolonged allotransplant survival compared with HO-1-BM-MSCs and BM-MSCs alone due to the dual effects of MSC homing by CXCR3 and cytoprotection/immunomodulation by HO-1 [71].

**Table 1:** Summary of preconditioned MSCs applied in organ/tissue allotransplantation.

Year [Ref]	Species	Donor_Recipient	Allotransplant_Specifics	MSC Origin_Tissue Souce ^@^	Preconditioning Reagent_Duration	MSC Dose (Timing, POD) ^&^	Administration Route	Allotransplant Survival (Preconditioned MSCs vs. MSCs) (Days) ^#^
***Pretreatment***								
2019 [36]	Rat	DA_LEW	corneal	Syn_BM	TNF-α and IL-1β_72 hr	1 × 10^6^ (1, 7)	IV_tail	no SD
2019 [37]	Rat	BN_LEW	VCA_hindlimb	Allo_BM	IFN-γ_24 hr	2 × 10^6^ (0)	IV_tail	15 vs. 10
2018 [38]	Mouse	Balb/c_C57BL/6	skin	Allo_BM	IL-17_5 days	NS (0)	IV_tail	19.2 vs. 15.8
2020 [40]	Mouse	C57BL/6_Balb/c	corneal	Syn_BM	TGF-β_72 hr	1 × 10^6^ (1, 7)	IV_tail	34.3 vs. 20.4
2020 [42]	Mouse	Balb/c_C57BL/6	heart	Syn_AD	poly(I:C)_1 hr	5 × 10^5^ (1)	IV_caudal	12.3 vs. 10.2
2018 [44]	Rat	Wistar_SD	kidney	Syn_BM	EPO_48 hr	1 × 10^6^ (0)	IV_caudal	no SD
***Gene Transfection***								
2014 [45]	Rat	DA_LEW	liver	Allo_BM	IL-10	2.5 × 10^5^ (0)	IV_jagular	76 vs. 66
2020 [46]	Rat	Wistar_LEW	corneal	Allo_BM	IL-10	2 × 10^6^ (0)	subconjunctival	28 vs. 12
2016 [47]	Rat	DA_LEW	liver	Syn_BM	hTGF-β1	5 × 10^6^ (0)	IV_protal	110 vs. 56.3
2020 [48]	Rat	Wistar_SD	heart	Syn_BM	IDO	1 × 10^6^ (2)	IV	SD
2015 [49]	Rabbit	New Zealand_Japanese White	kidney	Allo_BM	IDO	2 × 10^6^/Kg (0)	IV	62.8 vs. 16
2017 [52]	Rat	LEW_BN	liver_50% reduced size	Syn_BM	HO-1	1 × 10^7^ (0)	IV_penile	38 vs. 25
2016 [53]	Rat	LEW_BN	liver	Syn_BM	HO-1	5 × 10^6^ (0)	IV_penile	77 vs. 61
2016 [54]	Rat	BN_LEW	intestine	Syn_BM	HO-1	1 × 10^6^ (0)	IV_penile	24 vs. 15
2009 [59]	Mouse	C57BL/6_Balb/c	skin	Allo_BM	hHGF	1 × 10^6^ (0)	IV	16.73 vs. 14.27
2017 [61]	Rat	BN_LEW	kidney	Syn_AD	OX40Ig	2 × 10^6^ (−4)	IV_penile	14.2 vs. 10.2
2018 [62]	Rat	Wistar_SD	liver	Syn_BM	PD-L1Ig	NS (0)	IV_protal	100 vs. 23.4
2015 [63]	Rat	LEW_ACI	liver	Allo_BM	FoxP3	2.5 × 10^6^ (0)	IV_portal	>100 vs. 21
2019 [66]	Mouse	Balb/c_C57BL/6	heart	Syn_AD	IL-35	1 × 10^6^ (1)	IV_caudal	17.5 vs. 10.67
2020 [68]	Mouse	Balb/c_C57BL/6	heart	Syn_AD	sFgl2	1 × 10^6^ (1)	IV	52 vs. 15.3
2019 [69]	Rat	BN_LEW	VCA_ inferior epigastric flap	Xeno(human)_AD	CCR7	2 × 10^6^ (−1)	IV_tail	14.38 vs. 7.75
2013 [70]	Rat	Wistar_SD	kidney	Syn_BM	CXCR4	2 × 10^6^ (1)	IV	NR
2017 [71]	Rat	BN_LEW	intestine	Syn_BM	CXCR3/HO-1	5 × 10^6^ (−7)	IV	53 vs. 26

^@^: Allo, Syn: MSCs isolated from donor, recipient strain, respectively; Xeno: MSCs derived from xenogenic origin; BM: bone marrow; AD: adipose tissue; ^&^: NS: not specified; ^#^: SD: significant differences; NR: not reported, all other data showed statistically significant differences.

## 4. Discussion

**Figure 2:**
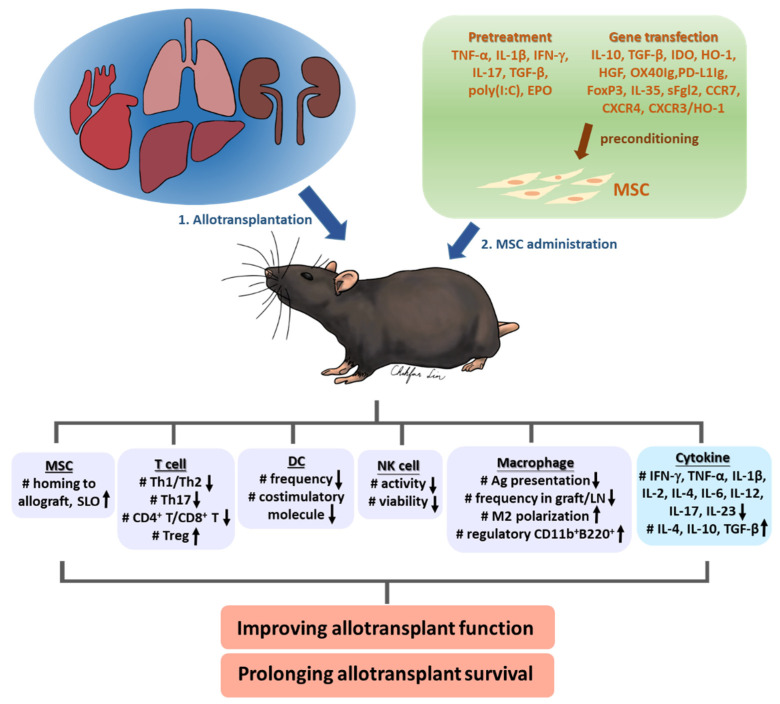
Summary of in vivo effects of preconditioned MSCs applied in allotransplantation.

Based on the reports summarized above, most types of preconditioned MSCs significantly prolonged allotransplant survival, but the effects varied. Preconditioned MSCs may affect allotransplantation outcomes by modulating cytokines, Tregs, Th1/Th2/Th17 cells, DCs, NK cells, and macrophages (Figure 2). Among these, the increase in Tregs and anti-inflammatory cytokines IL-10 and TGF-β was the most observed. When the reports on the same transplant are grouped together (Table 2), we find that besides the characteristics of the transplant, homing of the preconditioned MSCs is an important factor. As shown in Table 1 and Table 2, IV administration was the most chosen route for MSCs. However, while portal vein infusion directed MSCs to the liver, other forms of IV administration led to accumulation of MSC in the lung capillary bed due to their large cell size [24,72], and only a small proportion of cells was migrated to tissues. Promoting the cell homing to transplant would help to improve the transplantation outcome. For example, portal vein-administered preconditioned MSCs had better efficacy in promoting liver transplant survival and even induced donor-specific tolerance [63], in comparison with the cells that were administered systemically. Furthermore, He et al. administered superparamagnetic iron oxide-labeled IDO-BM-MSCs to the recipients with a magnet on their back and observed the cells migrated to the graft, which may also contribute to the significant improvement of transplanted kidney survival and the induced donor-specific tolerance [49]. Transfection of the chemokine receptor genes helped in targeted migration of MSCs to the SLOs or the transplants and exerted immunomodulatory functions. Yin et al. showed the superiority of CXCR3/HO-1-BM-MSCs to HO-1-BM-MSCs in promoting allograft survival, suppressing NK activity, reducing apoptosis and proinflammatory cytokines, and inducing Treg generation and anti-inflammatory cytokines. Furthermore, induced expression of CXCR4 and higher intragraft levels of MSCs resulted from certain preconditioning treatments, such as EPO pretreatment and TGF-β1 transfection [44,47]. These data support the notion that facilitating MSC homing would be beneficial for allotransplantation outcomes. Practically, the approach to transfect two genes and simultaneously targeted MSC homing and immunomodulation provides great potential.

MSCs could also be administered through the intra-arterial (IA) route. Although this route is more invasive and less commonly applied than the IV route, more MSCs were shown to migrate to the peripheral tissues, as illustrated in the studies on kidney injury and cerebral ischemia [72,73,74]. Direct injection to the target tissue has been reported in endocardial [75], intra-articular [76], and intracerebral [77] administration, although the efficacy may be compromised by the lower oxygen supply within the tissues such that the cell survival rate was extremely low [78]. In the transplantation field, the time between organ/tissue procurement and transplantation surgery provides a unique window to pretreat the transplant ex vivo, which can be regarded as an alternative way of local administration. In a kidney autotransplantation model, MSCs added during the normothermic machine perfusion stage can be retained within the kidney cortex for 14 days [79]. Pieróg et al. infused the lung graft with hIL-10-BM-MSCs prior to implantation and found a significant improvement in the lung function on POD 5 [80]. The hindlimb pre-treated ex vivo VCA with 5% O_2_ (hypoxia)-preconditioned BM-MSC significantly prolonged VCA survival [81,82]. MSCs were shown to distribute in the perivascular space within the graft and significant enhancement of IDO and reduction in pro-inflammatory intercellular adhesion molecule 1 (ICAM-1), monocyte chemoattractant protein-1 (MCP-1), and C-X-C motif chemokine ligand 9 (CXCL9) expression was observed in the transplant, which suggested that localized instead of systemic effects were exerted by the MSCs.

Currently, long-term consistent intake of immunosuppressants is required to maintain allotransplant survival. However, all studies discussed in this report only evaluated the effects of preconditioned MSCs without supplemented immunosuppressants. The commonly used immunosuppressants, such as cyclosporin (CsA), tacrolimus, rapamycin, and mycophenolate mofetil, have different impacts on the level or function of specific immune cell populations, such as Tregs [83,84], or the interactions between different types of immune cells [85]. On the other hand, CsA was shown to promote MSC survival and exerted additive immunomodulatory effects on MSCs in vivo [86], although rapamycin may affect MSC proliferation [87]. In reverse, MSCs also modulate the function of the immunosuppressants [87,88]. The data that combination of short-term immunosuppressant regimen and MSCs significantly prolong allotransplant survival or even induce donor-specific tolerance demonstrated a synergistic effect [12,14,89,90]. While many studies listed in the current review have reported the advantages of preconditioned MSCs, it is worthwhile to explore appropriate supplementation of low-dose or short-term immunosuppressants to maximize the synergistic effects and further improve the transplantation outcome.

## 5. Conclusions

In summary, MSC preconditioning by pretreatments or gene transfection holds great potential in improving the allotransplantation outcomes. Individual preconditioning may enhance the immunomodulatory and/or homing capacities of MSCs. Further optimization of the preconditioned MSCs efficacy can be through transfection of multiple genes, IA administration, or ex vivo-treatment of the allograft before implantation. Furthermore, appropriate incorporation of short-term or low-dose of traditional immunosuppressants may exert synergistic effects on the preconditioned MSCs to improve allotransplantation outcomes or even induce donor-specific tolerance.

## Figures and Tables

**Table 2:** Preconditioned MSCs applied in organ/tissue allotransplantation, grouped by transplant type.

Year [Ref]	Species	Allotransplant_Specifics	MSC Origin_Tissue Souce ^@^	Preconditioning Reagent_Duration	MSC Dose (Timing, POD) ^&^	Administration Route	Allotransplant Survival (Preconditioned MSCs vs. MSCs) (Days) ^#^
2017 [52]	Rat	liver_50% reduced size	Syn_BM	HO-1	1 × 10^7^ (0)	IV_penile	38 vs. 25
2016 [53]	Rat	liver	Syn_BM	HO-1	5 × 10^6^ (0)	IV_penile	77 vs. 61
2014 [45]	Rat	liver	Allo_BM	IL-10	2.5 × 10^5^ (0)	IV_jagular	76 vs. 66
2016 [47]	Rat	liver	Syn_BM	hTGF-β1	5 × 10^6^ (0)	IV_portal	110 vs. 56.3
2018 [62]	Rat	liver	Syn_BM	PD-L1Ig	NS (0)	IV_portal	100 vs. 23.4
2015 [63]	Rat	liver	Allo_BM	FoxP3	2.5 × 10^6^ (0)	IV_portal	>100 vs. 21
2020 [42]	Mouse	heart	Syn_AD	poly(I:C)_1 hr	5 × 10^5^ (1)	IV_caudal	12.3 vs. 10.2
2020 [48]	Rat	heart	Syn_BM	IDO	1 × 10^6^ (2)	IV	SD
2019 [66]	Mouse	heart	Syn_AD	IL-35	1 × 10^6^ (1)	IV_caudal	17.5 vs. 10.67
2020 [68]	Mouse	heart	Syn_AD	sFgl2	1 × 10^6^ (1)	IV	52 vs. 15.3
2018 [44]	Rat	kidney	Syn_BM	EPO_48 hr	1 × 10^6^ (0)	IV_caudal	no SD
2015 [49]	Rabbit	kidney	Allo_BM	IDO	2 × 10^6^/Kg (0)	IV	62.8 vs. 16
2017 [61]	Rat	kidney	Syn_AD	OX40Ig	2 × 10^6^ (−4)	IV_penile	14.2 vs. 10.2
2013 [70]	Rat	kidney	Syn_BM	CXCR4	2 × 10^6^ (1)	IV	NR
2016 [54]	Rat	intestine	Syn_BM	HO-1	1 × 10^6^ (0)	IV_penile	24 vs. 15
2017 [71]	Rat	intestine	Syn_BM	CXCR3 and HO-1	5 × 10^6^ (−7)	IV	53 vs. 26
2019 [36]	Rat	corneal	Syn_BM	TNF-α and IL-1β_72 hr	1 × 10^6^ (1, 7)	IV_tail	no SD
2020 [40]	Mouse	corneal	Syn_BM	TGF-β_72 hr	1 × 10^6^ (1, 7)	IV_tail	34.3 vs. 20.4
2020 [46]	Rat	corneal	Allo_BM	IL-10	2 × 10^6^ (0)	subconjunctival	28 vs. 12
2019 [37]	Rat	VCA_hindlimb	Allo_BM	IFN-γ_24 hr	2 × 10^6^ (0)	IV_tail	15 vs. 10
2019 [69]	Rat	VCA_inferior epigastric flap	Xeno(human)_AD	CCR7	2 × 10^6^ (−1)	IV_tail	14.38 vs. 7.75
2018 [38]	Mouse	skin	Allo_BM	IL-17_5 days	NS (0)	IV_tail	19.2 vs. 15.8
2009 [59]	Mouse	skin	Allo_BM	hHGF	1 × 10^6^ (0)	IV	16.73 vs. 14.27

^@^: Allo, Syn: MSCs isolated from donor, recipient strain, respectively; Xeno: MSCs derived from xenogenic origin; BM: bone marrow; AD: adipose tissue; ^&^: NS: not specified; ^#^: SD: significant differences; NR: not reported, all other data showed statistically significant differences.

## References

[1] Gorantla V.S., Barker J.H., Jones J.W., Prabhune K., Maldonado C., Granger D.K. (2000). Immunosuppressive agents in transplantation: Mechanisms of action and current anti-rejection strategies. Microsurgery.

[2] Hoogduijn M.J., Issa F., Casiraghi F., Reinders M.E.J. (2021). Cellular therapies in organ transplantation. Transpl. Int. Off. J. Eur. Soc. Organ Transplant..

[3] Viswanathan S., Shi Y., Galipeau J., Krampera M., Leblanc K., Martin I., Nolta J., Phinney D.G., Sensebe L. (2019). Mesenchymal stem versus stromal cells: International Society for Cell & Gene Therapy (ISCT^®^) Mesenchymal Stromal Cell committee position statement on nomenclature. Cytotherapy.

[4] Da Silva Meirelles L., Chagastelles P.C., Nardi N.B. (2006). Mesenchymal stem cells reside in virtually all post-natal organs and tissues. J. Cell Sci..

[5] Cheng H.Y., Ghetu N., Wallace C.G., Wei F.C., Liao S.K. (2014). The impact of mesenchymal stem cell source on proliferation, differentiation, immunomodulation and therapeutic efficacy. J. Stem Cell Res. Ther..

[6] Chen P.M., Yen M.L., Liu K.J., Sytwu H.K., Yen B.L. (2011). Immunomodulatory properties of human adult and fetal multipotent mesenchymal stem cells. J. Biomed. Sci..

[7] Caplan A.I., Dennis J.E. (2006). Mesenchymal stem cells as trophic mediators. J. Cell. Biochem..

[8] Khubutiya M.S., Vagabov A.V., Temnov A.A., Sklifas A.N. (2014). Paracrine mechanisms of proliferative, anti-apoptotic and anti-inflammatory effects of mesenchymal stromal cells in models of acute organ injury. Cytotherapy.

[9] Mohammadalipour A., Dumbali S.P., Wenzel P.L. (2020). Mitochondrial Transfer and Regulators of Mesenchymal Stromal Cell Function and Therapeutic Efficacy. Front. Cell Dev. Biol..

[10] Court A.C., Le-Gatt A., Luz-Crawford P., Parra E., Aliaga-Tobar V., Bátiz L.F., Contreras R.A., Ortúzar M.I., Kurte M., Elizondo-Vega R. (2020). Mitochondrial transfer from MSCs to T cells induces Treg differentiation and restricts inflammatory response. EMBO Rep..

[11] Do J.S., Zwick D., Kenyon J.D., Zhong F., Askew D., Huang A.Y., Van’t Hof W., Finney M., Laughlin M.J. (2021). Mesenchymal stromal cell mitochondrial transfer to human induced T-regulatory cells mediates FOXP3 stability. Sci. Rep..

[12] Cheng H.Y., Ghetu N., Huang W.C., Wang Y.L., Wallace C.G., Wen C.J., Chen H.C., Shih L.Y., Lin C.F., Hwang S.M. (2014). Syngeneic adipose-derived stem cells with short-term immunosuppression induce vascularized composite allotransplantation tolerance in rats. Cytotherapy.

[13] Ge W., Jiang J., Arp J., Liu W., Garcia B., Wang H. (2010). Regulatory T-cell generation and kidney allograft tolerance induced by mesenchymal stem cells associated with indoleamine 2,3-dioxygenase expression. Transplantation.

[14] Ge W., Jiang J., Baroja M.L., Arp J., Zassoko R., Liu W., Bartholomew A., Garcia B., Wang H. (2009). Infusion of mesenchymal stem cells and rapamycin synergize to attenuate alloimmune responses and promote cardiac allograft tolerance. Am. J. Transplant..

[15] Shi M., Liu Z., Wang Y., Xu R., Sun Y., Zhang M., Yu X., Wang H., Meng L., Su H. (2017). A Pilot Study of Mesenchymal Stem Cell Therapy for Acute Liver Allograft Rejection. Stem Cells Transl. Med..

[16] Keller C.A., Gonwa T.A., Hodge D.O., Hei D.J., Centanni J.M., Zubair A.C. (2018). Feasibility, Safety, and Tolerance of Mesenchymal Stem Cell Therapy for Obstructive Chronic Lung Allograft Dysfunction. Stem Cells Transl. Med..

[17] Erpicum P., Weekers L., Detry O., Bonvoisin C., Delbouille M.H., Grégoire C., Baudoux E., Briquet A., Lechanteur C., Maggipinto G. (2019). Infusion of third-party mesenchymal stromal cells after kidney transplantation: A phase I-II, open-label, clinical study. Kidney Int..

[18] Perico N., Casiraghi F., Introna M., Gotti E., Todeschini M., Cavinato R.A., Capelli C., Rambaldi A., Cassis P., Rizzo P. (2011). Autologous mesenchymal stromal cells and kidney transplantation: A pilot study of safety and clinical feasibility. Clin. J. Am. Soc. Nephrol..

[19] Reinders M.E., de Fijter J.W., Roelofs H., Bajema I.M., de Vries D.K., Schaapherder A.F., Claas F.H., van Miert P.P., Roelen D.L., van Kooten C. (2013). Autologous bone marrow-derived mesenchymal stromal cells for the treatment of allograft rejection after renal transplantation: Results of a phase I study. Stem Cells Transl. Med..

[20] Sun Q., Huang Z., Han F., Zhao M., Cao R., Zhao D., Hong L., Na N., Li H., Miao B. (2018). Allogeneic mesenchymal stem cells as induction therapy are safe and feasible in renal allografts: Pilot results of a multicenter randomized controlled trial. J. Transl. Med..

[21] Tan J., Wu W., Xu X., Liao L., Zheng F., Messinger S., Sun X., Chen J., Yang S., Cai J. (2012). Induction therapy with autologous mesenchymal stem cells in living-related kidney transplants: A randomized controlled trial. JAMA.

[22] Reinders M.E.J., Groeneweg K.E., Hendriks S.H., Bank J.R., Dreyer G.J., de Vries A.P.J., van Pel M., Roelofs H., Huurman V.A.L., Meij P. (2021). Autologous bone marrow-derived mesenchymal stromal cell therapy with early tacrolimus withdrawal: The randomized prospective, single-center, open-label TRITON study. Am. J. Transplant..

[23] Toma C., Pittenger M.F., Cahill K.S., Byrne B.J., Kessler P.D. (2002). Human mesenchymal stem cells differentiate to a cardiomyocyte phenotype in the adult murine heart. Circulation.

[24] Lee R.H., Pulin A.A., Seo M.J., Kota D.J., Ylostalo J., Larson B.L., Semprun-Prieto L., Delafontaine P., Prockop D.J. (2009). Intravenous hMSCs improve myocardial infarction in mice because cells embolized in lung are activated to secrete the anti-inflammatory protein TSG-6. Cell Stem Cell.

[25] Gholamrezanezhad A., Mirpour S., Bagheri M., Mohamadnejad M., Alimoghaddam K., Abdolahzadeh L., Saghari M., Malekzadeh R. (2011). In vivo tracking of 111In-oxine labeled mesenchymal stem cells following infusion in patients with advanced cirrhosis. Nucl. Med. Biol..

[26] Nitzsche F., Müller C., Lukomska B., Jolkkonen J., Deten A., Boltze J. (2017). Concise Review: MSC Adhesion Cascade-Insights into Homing and Transendothelial Migration. Stem Cells.

[27] Yuan X., Logan T.M., Ma T. (2019). Metabolism in Human Mesenchymal Stromal Cells: A Missing Link Between hMSC Biomanufacturing and Therapy?. Front. Immunol..

[28] Mangi A.A., Noiseux N., Kong D., He H., Rezvani M., Ingwall J.S., Dzau V.J. (2003). Mesenchymal stem cells modified with Akt prevent remodeling and restore performance of infarcted hearts. Nat. Med..

[29] Baldari S., Di Rocco G., Piccoli M., Pozzobon M., Muraca M., Toietta G. (2017). Challenges and Strategies for Improving the Regenerative Effects of Mesenchymal Stromal Cell-Based Therapies. Int. J. Mol. Sci..

[30] Seo Y., Shin T.H., Kim H.S. (2019). Current Strategies to Enhance Adipose Stem Cell Function: An Update. Int. J. Mol. Sci..

[31] Xie X., Sun A., Zhu W., Huang Z., Hu X., Jia J., Zou Y., Ge J. (2012). Transplantation of Mesenchymal Stem Cells Preconditioned with Hydrogen Sulfide Enhances Repair of Myocardial Infarction in Rats. Tohoku J. Exp. Med..

[32] Wang J.W., Qiu Y.R., Fu Y., Liu J., He Z.J., Huang Z.T. (2017). Transplantation with hypoxia-preconditioned mesenchymal stem cells suppresses brain injury caused by cardiac arrest-induced global cerebral ischemia in rats. J. Neurosci. Res..

[33] Nan Z., Fan H., Tang Q., Zhang M., Xu M., Chen Q., Liu Y., Dong Y., Wu H., Deng S. (2018). Dual expression of CXCR4 and IL-35 enhances the therapeutic effects of BMSCs on TNBS-induced colitis in rats through expansion of Tregs and suppression of Th17 cells. Biochem. Biophys. Res. Commun..

[34] Tang B., Li X., Liu Y., Chen X., Li X., Chu Y., Zhu H., Liu W., Xu F., Zhou F. (2018). The Therapeutic Effect of ICAM-1-Overexpressing Mesenchymal Stem Cells on Acute Graft-Versus-Host Disease. Cell. Physiol. Biochem..

[35] Krampera M., Cosmi L., Angeli R., Pasini A., Liotta F., Andreini A., Santarlasci V., Mazzinghi B., Pizzolo G., Vinante F. (2006). Role for interferon-gamma in the immunomodulatory activity of human bone marrow mesenchymal stem cells. Stem Cells.

[36] Murphy N., Treacy O., Lynch K., Morcos M., Lohan P., Howard L., Fahy G., Griffin M.D., Ryan A.E., Ritter T. (2019). TNF-α/IL-1β—licensed mesenchymal stromal cells promote corneal allograft survival via myeloid cell-mediated induction of Foxp3+ regulatory T cells in the lung. FASEB J..

[37] Wang Y., Xi Y., Han F., Liu Y., Li N., Ren Z., Xue J., Guo L., Hu D. (2019). Vascularized composite allograft rejection is delayed by infusion of IFN-γ-conditioned BMSCs through upregulating PD-L1. Cell Tissue Res..

[38] Ma T., Wang X., Jiao Y., Wang H., Qi Y., Gong H., Zhang L., Jiang D. (2018). Interleukin 17 (IL-17)-Induced Mesenchymal Stem Cells Prolong the Survival of Allogeneic Skin Grafts. Ann. Transplant..

[39] Rodríguez T.M., Saldías A., Irigo M., Zamora J.V., Perone M.J., Dewey R.A. (2015). Effect of TGF-β1 Stimulation on the Secretome of Human Adipose-Derived Mesenchymal Stromal Cells. Stem Cells Transl. Med..

[40] Lynch K., Treacy O., Chen X., Murphy N., Lohan P., Islam M.N., Donohoe E., Griffin M.D., Watson L., McLoughlin S. (2020). TGF-β1-Licensed Murine MSCs Show Superior Therapeutic Efficacy in Modulating Corneal Allograft Immune Rejection In Vivo. Mol. Ther..

[41] Shoshina O.O., Kozhin P.M., Shadrin V.S., Romashin D.D., Rusanov A.L., Luzgina N.G. (2021). Phenotypic Features of Mesenchymal Stem Cell Subpopulations Obtained under the Influence of Various Toll-Like Receptors Ligands. Bull. Exp. Biol. Med..

[42] Bao Z., Li J., Zhang P., Pan Q., Liu B., Zhu J., Jian Q., Jia D., Yi C., Moeller C.J. (2020). Toll-Like Receptor 3 Activator Preconditioning Enhances Modulatory Function of Adipose—Derived Mesenchymal Stem Cells in a Fully MHC-Mismatched Murine Model of Heterotopic Heart Transplantation. Ann. Transplant..

[43] Purroy C., Fairchild R.L., Tanaka T., Baldwin W.M., Manrique J., Madsen J.C., Colvin R.B., Alessandrini A., Blazar B.R., Fribourg M. (2017). Erythropoietin receptor-mediated molecular crosstalk promotes T cell immunoregulation and transplant survival. J. Am. Soc. Nephrol..

[44] Zhang Y., Zhou S., Hu J.M., Chen H., Liu D., Li M., Guo Y., Fan L.P., Li L.Y., Liu Y.G. (2018). Preliminary Study of Bone Marrow-Derived Mesenchymal Stem Cells Pretreatment with Erythropoietin in Preventing Acute Rejection after Rat Renal Transplantation. Transplant. Proc..

[45] Niu J., Yue W., Song Y., Zhang Y., Qi X., Wang Z., Liu B., Shen H., Hu X. (2014). Prevention of acute liver allograft rejection by IL-10-engineered mesenchymal stem cells. Clin. Exp. Immunol..

[46] Lu X., Ru Y., Chu C., Lv Y., Gao Y., Jia Z., Huang Y., Zhang Y., Zhao S. (2020). Lentivirus-mediated IL-10-expressing Bone Marrow Mesenchymal Stem Cells promote corneal allograft survival via upregulating lncRNA 003946 in a rat model of corneal allograft rejection. Theranostics.

[47] Tang J., Yang R., Lv L., Yao A., Pu L., Yin A., Li X., Yu Y., Nyberg S.L., Wang X. (2016). Transforming growth factor-β-Expressing Mesenchymal Stem Cells Induce Local Tolerance in a Rat Liver Transplantation Model of Acute Rejection. Stem Cells.

[48] He J.G., Li B.B., Zhou L., Yan D., Xie Q.L., Zhao W. (2020). Indoleamine 2,3-dioxgenase-transfected mesenchymal stem cells suppress heart allograft rejection by increasing the production and activity of dendritic cells and regulatory T cells. J. Investig. Med..

[49] He Y., Zhou S., Liu H., Shen B., Zhao H., Peng K., Wu X. (2015). Indoleamine 2, 3-Dioxgenase Transfected Mesenchymal Stem Cells Induce Kidney Allograft Tolerance by Increasing the Production and Function of Regulatory T Cells. Transplantation.

[50] Verheij M., Zeerleder S., Voermans C. (2021). Heme oxygenase-1: Equally important in allogeneic hematopoietic stem cell transplantation and organ transplantation?. Transpl. Immunol..

[51] Chabannes D., Hill M., Merieau E., Rossignol J., Brion R., Soulillou J.P., Anegon I., Cuturi M.C. (2007). A role for heme oxygenase-1 in the immunosuppressive effect of adult rat and human mesenchymal stem cells. Blood.

[52] Shen Z.Y., Wu B., Liu T., Yang Y., Yin M.L., Zheng W.P., Zhang B.Y., Song H.L. (2017). Immunomodulatory effects of bone marrow mesenchymal stem cells overexpressing heme oxygenase-1: Protective effects on acute rejection following reduced-size liver transplantation in a rat model. Cell. Immunol..

[53] Wu B., Song H.L., Yang Y., Yin M.L., Zhang B.Y., Cao Y., Dong C., Shen Z.Y. (2016). Improvement of Liver Transplantation Outcome by Heme Oxygenase-1-Transduced Bone Marrow Mesenchymal Stem Cells in Rats. Stem Cells Int..

[54] Yang Y., Song H.L., Zhang W., Wu B.J., Fu N.N., Dong C., Shen Z.Y. (2016). Heme oxygenase-1-transduced bone marrow mesenchymal stem cells in reducing acute rejection and improving small bowel transplantation outcomes in rats. Stem Cell Res. Ther..

[55] Okunishi K., Dohi M., Nakagome K., Tanaka R., Mizuno S., Matsumoto K., Miyazaki J., Nakamura T., Yamamoto K. (2005). A novel role of hepatocyte growth factor as an immune regulator through suppressing dendritic cell function. J. Immunol..

[56] Chen Q.-H., Wu F., Liu L., Chen H.-b., Zheng R.-Q., Wang H.-L., Yu L.-N. (2020). Mesenchymal stem cells regulate the Th17/Treg cell balance partly through hepatocyte growth factor in vitro. Stem Cell Res. Ther..

[57] Lu Z., Chang W., Meng S., Xu X., Xie J., Guo F., Yang Y., Qiu H., Liu L. (2019). Mesenchymal stem cells induce dendritic cell immune tolerance via paracrine hepatocyte growth factor to alleviate acute lung injury. Stem Cell Res. Ther..

[58] Duan H.F., Wu C.T., Wu D.L., Lu Y., Liu H.J., Ha X.Q., Zhang Q.W., Wang H., Jia X.X., Wang L.S. (2003). Treatment of myocardial ischemia with bone marrow-derived mesenchymal stem cells overexpressing hepatocyte growth factor. Mol. Ther..

[59] Bian L., Guo Z.K., Wang H.X., Wang J.S., Wang H., Li Q.F., Yang Y.F., Xiao F.J., Wu C.T., Wang L.S. (2009). In vitro and in vivo immunosuppressive characteristics of hepatocyte growth factor-modified murine mesenchymal stem cells. In Vivo.

[60] Lin C.H., Wang Y.L., Anggelia M.R., Chuang W.Y., Cheng H.Y., Mao Q., Zelken J.A., Lin C.H., Zheng X.X., Lee W.P. (2016). Combined Anti-CD154/CTLA4Ig Costimulation Blockade-Based Therapy Induces Donor-Specific Tolerance to Vascularized Osteomyocutaneous Allografts. Am. J. Transplant..

[61] Liu T., Zhang Y., Shen Z., Zou X., Chen X., Chen L., Wang Y. (2017). Immunomodulatory effects of OX40Ig gene-modified adipose tissue-derived mesenchymal stem cells on rat kidney transplantation. Int. J. Mol. Med..

[62] Li P., Zhang Y.Y., Deng J. (2018). PDL1Ig gene-loaded BMSCs Induce liver transplantation immune tolerance. Eur. Rev. Med Pharmacol. Sci..

[63] Qi H., Chen G., Huang Y., Si Z., Li J. (2015). Foxp3-modified bone marrow mesenchymal stem cells promotes liver allograft tolerance through the generation of regulatory T cells in rats. J. Transl. Med..

[64] Liu X.G., Liu Y., Chen F. (2017). Soluble fibrinogen like protein 2 (sFGL2), the novel effector molecule for immunoregulation. Oncotarget.

[65] Collison L.W., Chaturvedi V., Henderson A.L., Giacomin P.R., Guy C., Bankoti J., Finkelstein D., Forbes K., Workman C.J., Brown S.A. (2010). IL-35-mediated induction of a potent regulatory T cell population. Nat. Immunol..

[66] Wang W., Zhao N., Li B., Gao H., Yan Y., Guo H. (2019). Inhibition of cardiac allograft rejection in mice using interleukin-35-modified mesenchymal stem cells. Scand. J. Immunol..

[67] Pan G., Zhao Z., Tang C., Ding L., Li Z., Zheng D., Zong L., Wu Z. (2018). Soluble fibrinogen-like protein 2 ameliorates acute rejection of liver transplantation in rat via inducing Kupffer cells M2 polarization. Cancer Med..

[68] Gao C., Wang X., Lu J., Li Z., Jia H., Chen M., Chang Y., Liu Y., Li P., Zhang B. (2020). Mesenchymal stem cells transfected with sFgl2 inhibit the acute rejection of heart transplantation in mice by regulating macrophage activation. Stem Cell Res. Ther..

[69] Ma T., Luan S., Tao R., Lu D., Guo L., Liu J., Shu J., Zhou X., Han Y., Jia Y. (2019). Targeted Migration of Human Adipose-Derived Stem Cells to Secondary Lymphoid Organs Enhances Their Immunomodulatory Effect and Prolongs the Survival of Allografted Vascularized Composites. Stem Cells.

[70] Cao Z., Zhang G., Wang F., Liu H., Liu L., Han Y., Zhang J., Yuan J. (2013). Protective effects of mesenchymal stem cells with CXCR4 up-regulation in a rat renal transplantation model. PLoS ONE.

[71] Yin M.L., Song H.L., Yang Y., Zheng W.P., Liu T., Shen Z.Y. (2017). Effect of CXCR3/HO-1 genes modified bone marrow mesenchymal stem cells on small bowel transplant rejection. World J. Gastroenterol..

[72] Leibacher J., Henschler R. (2016). Biodistribution, migration and homing of systemically applied mesenchymal stem/stromal cells. Stem Cell Res. Ther..

[73] Tögel F., Yang Y., Zhang P., Hu Z., Westenfelder C. (2008). Bioluminescence imaging to monitor the in vivo distribution of administered mesenchymal stem cells in acute kidney injury. Am. J. Physiol. Ren. Physiol..

[74] Walczak P., Zhang J., Gilad A.A., Kedziorek D.A., Ruiz-Cabello J., Young R.G., Pittenger M.F., van Zijl P.C., Huang J., Bulte J.W. (2008). Dual-modality monitoring of targeted intraarterial delivery of mesenchymal stem cells after transient ischemia. Stroke.

[75] Freyman T., Polin G., Osman H., Crary J., Lu M., Cheng L., Palasis M., Wilensky R.L. (2006). A quantitative, randomized study evaluating three methods of mesenchymal stem cell delivery following myocardial infarction. Eur. Heart J..

[76] Geng Y., Chen J., Alahdal M., Chang C., Duan L., Zhu W., Mou L., Xiong J., Wang M., Wang D. (2020). Intra-articular injection of hUC-MSCs expressing miR-140-5p induces cartilage self-repairing in the rat osteoarthritis. J. Bone Miner. Metab..

[77] Mohme M., Maire C.L., Geumann U., Schliffke S., Dührsen L., Fita K., Akyüz N., Binder M., Westphal M., Guenther C. (2020). Local Intracerebral Immunomodulation Using Interleukin-Expressing Mesenchymal Stem Cells in Glioblastoma. Clin. Cancer Res..

[78] McGinley L.M., McMahon J., Stocca A., Duffy A., Flynn A., O’Toole D., O’Brien T. (2013). Mesenchymal stem cell survival in the infarcted heart is enhanced by lentivirus vector-mediated heat shock protein 27 expression. Hum. Gene Ther..

[79] Lohmann S., Pool M.B.F., Rozenberg K.M., Keller A.K., Moers C., Møldrup U., Møller B.K., Lignell S.J.M., Krag S., Sierra-Parraga J.M. (2020). Mesenchymal stromal cell treatment of donor kidneys during ex vivo normothermic machine perfusion: A porcine renal autotransplantation study. Am. J. Transplant..

[80] Pieróg J., Tamo L., Fakin R., Kocher G., Gugger M., Grodzki T., Geiser T., Gazdhar A., Schmid R.A. (2018). Bone marrow stem cells modified with human interleukin 10 attenuate acute rejection in rat lung allotransplantation. Eur. J. Cardio-Thorac. Surg..

[81] Soares M.A., Massie J.P., Rifkin W.J., Rao N., Duckworth A.M., Park C., Kadle R.L., David J.A., Rabbani P.S., Ceradini D.J. (2018). Ex vivo allotransplantation engineering: Delivery of mesenchymal stem cells prolongs rejection-free allograft survival. Am. J. Transplant..

[82] Wang Y., Wang S., Gu C., Xiong Y., Shen H., Liu F., Yang J. (2020). Ex-vivo treatment of allografts using adipose-derived stem cells induced prolonged rejection-free survival in an allogenic hind-limb transplantation model. Ann. Transl. Med..

[83] Gao W., Lu Y., El Essawy B., Oukka M., Kuchroo V.K., Strom T.B. (2007). Contrasting effects of cyclosporine and rapamycin in de novo generation of alloantigen-specific regulatory T cells. Am. J. Transplant..

[84] Lee J.U., Kim L.K., Choi J.M. (2018). Revisiting the Concept of Targeting NFAT to Control T Cell Immunity and Autoimmune Diseases. Front. Immunol..

[85] Steines L., Poth H., Schuster A., Amann K., Banas B., Bergler T. (2021). Disruption of Tfh:B Cell Interactions Prevents Antibody-Mediated Rejection in a Kidney Transplant Model in Rats: Impact of Calcineurin Inhibitor Dose. Front. Immunol..

[86] Hajkova M., Jaburek F., Porubska B., Bohacova P., Holan V., Krulova M. (2019). Cyclosporine A promotes the therapeutic effect of mesenchymal stem cells on transplantation reaction. Clin. Sci..

[87] Hoogduijn M.J., Crop M.J., Korevaar S.S., Peeters A.M., Eijken M., Maat L.P., Balk A.H., Weimar W., Baan C.C. (2008). Susceptibility of human mesenchymal stem cells to tacrolimus, mycophenolic acid, and rapamycin. Transplantation.

[88] Hajkova M., Hermankova B., Javorkova E., Bohacova P., Zajicova A., Holan V., Krulova M. (2017). Mesenchymal Stem Cells Attenuate the Adverse Effects of Immunosuppressive Drugs on Distinct T Cell Subopulations. Stem Cell Rev. Rep..

[89] Schweizer R., Taddeo A., Waldner M., Klein H.J., Fuchs N., Kamat P., Targosinski S., Barth A.A., Drach M.C., Gorantla V.S. (2020). Adipose-derived stromal cell therapy combined with a short course nonmyeloablative conditioning promotes long-term graft tolerance in vascularized composite allotransplantation. Am. J. Transplant..

[90] Eggenhofer E., Renner P., Soeder Y., Popp F.C., Hoogduijn M.J., Geissler E.K., Schlitt H.J., Dahlke M.H. (2011). Features of synergism between mesenchymal stem cells and immunosuppressive drugs in a murine heart transplantation model. Transpl. Immunol..

